# Correlations between MRI and Information Processing Speed in MS: A Meta-Analysis

**DOI:** 10.1155/2014/975803

**Published:** 2014-03-25

**Authors:** S. M. Rao, A. L. Martin, R. Huelin, E. Wissinger, Z. Khankhel, E. Kim, K. Fahrbach

**Affiliations:** ^1^Cleveland Clinic, 9500 Euclid Avenue, Cleveland, OH 44195, USA; ^2^Evidera, 420 Bedford Street, Lexington, MA 02420, USA; ^3^Novartis Pharmaceuticals Corporation, One Health Plaza, USEH 135-356, East Hanover, NJ 07936, USA

## Abstract

*Objectives*. To examine relationships between conventional MRI measures and the paced auditory serial addition test (PASAT) and symbol digit modalities test (SDMT). * Methods.* A systematic literature review was conducted. Included studies had ≥30 multiple sclerosis (MS) patients, administered the SDMT or PASAT, and measured T2LV or brain atrophy. Meta-analysis of MRI/information processing speed (IPS) correlations, analysis of MRI/IPS significance tests to account for reporting bias, and binomial testing to detect trends when comparing correlation strengths of SDMT versus PASAT and T2LV versus atrophy were conducted. * Results*. The 39 studies identified frequently reported only significant correlations, suggesting reporting bias. Direct meta-analysis was only feasible for correlations between SDMT and T2LV (*r* = −0.45, *P* < 0.001) and atrophy in patients with mixed-MS subtypes (*r* = −0.54, *P* < 0.001). Familywise Holm-Bonferroni testing found that selective reporting was not the source of at least half of significant results reported. Binomial tests (*P* = 0.006) favored SDMT over PASAT in strength of MRI correlations. * Conclusions*. A moderate-to-strong correlation exists between impaired IPS and MRI in mixed MS populations. Correlations with MRI were stronger for SDMT than for PASAT. Neither heterogeneity among populations nor reporting bias appeared to be responsible for these findings.

## 1. Introduction

Nearly half of multiple sclerosis (MS) patients exhibit impaired cognitive function [1] as assessed by standardized neuropsychological testing [2, 3]. One of the most common cognitive impairments involves information processing speed (IPS), occurring in 22%–25% of patients [3]. The paced auditory serial addition test (PASAT) is the most frequently administered test for assessing IPS in MS [3, 4]. In 1996, the PASAT was included as the sole cognitive measure in the MS functional composite (MSFC) [5–8], a performance-based clinical outcome measure used in MS clinical trials. Both the symbol digit modalities test (SDMT) and PASAT were historically included as part of the brief repeatable battery [9] and later in the Minimal Assessment of Cognitive Function in MS (MACFIMS) tool [10]. More recently, the Brief International Cognitive Assessment for MS (BICAMS) recommended use of the SDMT rather than the PASAT for measuring IPS [11]. After nearly two decades of experience, investigators and clinicians have expressed concerns regarding use of the PASAT because it is not well tolerated by patients and is prone to practice effects [12].

Recently, there has been some discussion of replacing the PASAT with the oral version of the SDMT as the cognitive component of the MSFC [13, 14]. In the most comprehensive comparison of the two measures conducted to date, Drake et al. [14] administered the SDMT and PASAT to 400 MS patients and 100 demographically matched controls; a subset of MS patients (*N* = 115) was retested 2.1 years later. The two tests were equally adept at discriminating MS patients from healthy controls based on a receiver operating characteristic (ROC) analysis. The test-retest correlations for the PASAT and SDMT were 0.78 and 0.74, respectively. No statistically significant differences were observed in changes of raw test scores over time (39.9 ± 13.5 to 41.9 ± 14.5 for the PASAT; 49.2 ± 11.8 to 48.9 ± 12.2 for the SDMT), suggesting that practice effects may be comparable. These data suggest that the PASAT and SDMT are at least equivalent in terms of sensitivity to IPS deficits in MS, reliability, and degree of practice effects. The SDMT has two major advantages: it is much better tolerated by patients and takes less time to administer (1.5 minutes for the SDMT; 3 minutes for the PASAT). A lingering question is whether the two measures exhibit comparable sensitivity to the underlying brain pathology that may give rise to IPS deficits.

Cognitive impairment is correlated with brain abnormalities as visualized by various magnetic resonance imaging (MRI) techniques [15]. Two of the most commonly derived MRI measures include T2-weighted lesion volume (T2LV) and whole-brain atrophy. As a consequence, there exists a large enough body of literature correlating the PASAT and SDMT with T2LV and atrophy to permit a meta-analysis. The primary goal of this study, therefore, was to determine which of the two IPS measures correlates more strongly with T2LV and atrophy based on a quantitative and qualitative review of the existing literature. A secondary goal was to determine whether T2LV or atrophy is the superior measure of brain pathology for understanding IPS dysfunction in MS.

## 2. Methods

A systematic search of the published literature evaluating MRI changes associated with cognitive outcomes in patients with MS was conducted in MEDLINE (via PubMed) and Embase. The search algorithms were limited to articles on human subjects published in English. There was no limit to the year of publication, and the search cut-off was December 1, 2011.

In addition to our review of indexed articles, conference proceedings from the most recent two years (2010 and 2011) were searched using keywords analogous to those used in MEDLINE and Embase. Conference proceedings from the following meetings were reviewed: Consortium of Multiple Sclerosis Centers (CMSC), European Committee for Treatment and Research in Multiple Sclerosis (ECTRIMS), American Committee for Treatment and Research in Multiple Sclerosis (ACTRIMS), and American Academy of Neurology (AAN).

To supplement the above searches and ensure optimal and complete literature retrieval, a manual check of the reference lists of recent systematic reviews and meta-analyses published in the past four years was performed.

Articles were selected for retrieval if they evaluated the use of conventional MRI techniques to report whole-brain measures, including either lesion volumes or counts, or atrophy and reported cognitive outcomes related to IPS. Only publications evaluating at least 30 adult patients with MS were included.

Data reporting correlations were extracted by a single investigator with validation by a second investigator. correlation coefficients (*r*-values), measures of statistical significance (*P* values), and mean cognitive scores were captured to evaluate the presence and strength of correlations between MRI measures and IPS performance. If a study stated evaluation of an outcome in the methods section but did not report on a relationship, the results were captured as not reported (NR). If the methods described only reporting significant results and did not report correlations, then data were extracted as not significant (NS).

Details on the cognitive tests also were captured and data were extracted separately for the PASAT 2- and 3-second tests. When correlations were reported between cognitive tests and multiple measures of atrophy, relationships to any whole-brain measure were captured.

Although we included studies assessing patients with any type of MS to evaluate how disease course may affect outcomes, we captured the proportion of patients with each subtype (relapsing-remitting, secondary progressive, primary progressive MS (PPMS), and progressive-relapsing) when reported. In studies where the MS subtype was not specified or patients with multiple subtypes were included, patients were categorized as having mixed MS subtypes.

A three-pronged approach was used to quantitatively analyze data. First, a meta-analysis of MRI/cognitive measures with near-complete data (>77% of studies reporting significant results) was conducted, imputing zero effects when there were missing data. Meta-analyses were conducted on the normalized correlations (i.e., using Fisher's *z* transformation), and the resulting estimates were back-transformed into Pearson correlations. (Note: Fisher's *z*s are roughly equivalent to Pearson correlations for *r* < 0.50 and are almost exactly the same for *r* < 0.30.)

The analyses were stratified by the MS subtypes reported in studies when sufficient data were available. The available data allowed stratifications for RRMS patients and patients with mixed MS subtypes. Optimally, meta-analyses would have been conducted for all measures and all strata, but missing data precluded this approach. However, meta-analyses were conducted, where feasible, to estimate the actual strength of the MRI/cognition relationship. The other prongs tested whether relationships existed but could not estimate the actual strength of those relationships.

The second set of analyses investigated whether significant effects reported between MRI and cognitive measures might be a product of reporting bias. Many studies investigate a large number of MRI and/or cognitive measures but only report results for the significant relationships. We used the Holm-Bonferroni method to determine the number of null hypotheses that could safely be rejected (while preserving a familywise error rate of 0.05) for any given combination of comparisons and MS patient populations [16]. Reject of a study's null hypothesis is rejection of the claim that there is no relationship between MRI measures and cognitive measures in that study. When conducting these procedures, we assumed that if a study did not report on a relationship, the result was not significant (e.g., when the authors of a paper mention they are looking at an outcome in the methods section and never report results or they state they will only report significant results).

The third set of analyses included a set of binomial tests to detect trends when comparing the SDMT to the PASAT and T2LV and atrophy. For instance, we investigated whether the relationship between the SDMT and T2LV was stronger than the relationship for the PASAT and T2LV across all studies reporting both an SDMT/T2LV and PASAT/T2LV relationship. If the relationship was equally strong, we would expect SDMT/T2LV correlations to be higher in 50% of studies and the PASAT/T2LV correlations to be higher in the other 50%. A preponderance of results in favor of one or the other measure suggests that it is more strongly correlated with the outcome of interest.

## 3. Results

**Figure 1:**
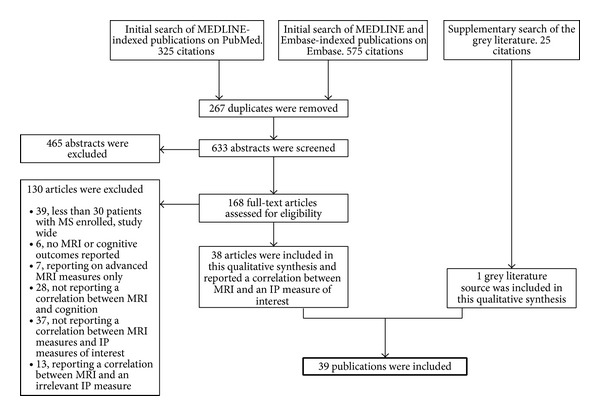
Flow chart for identification of studies in the systematic review.

The literature search identified 633 unique abstracts, which were assessed for potential inclusion. One-hundred sixty-eight abstracts were selected for retrieval and further assessment as full-text articles. Of those 168 articles, 130 studies were excluded during the full-text review as these publications did not meet the study inclusion criteria. Further details of study attrition are depicted in Figure 1. Thirty-nine studies reporting correlations between the PASAT and SDMT IPS measures and MRI assessments were identified for inclusion and analysis in this review [13, 17–54]. More studies evaluated the relationship between PASAT and atrophy (*n* = 24) [13, 18–21, 23–25, 27, 29–34, 37, 39, 42, 44, 47–49, 53, 54] or T2LV (*n* = 27) [13, 17–20, 24, 25, 27, 28, 30, 31, 33, 34, 36, 38–45, 47–50, 52] than SDMT and these MRI measures (*n* = 18 for both atrophy [13, 18–25, 27, 29, 30, 33, 34, 39, 47, 48, 54] and T2LV [13, 17–20, 22, 24, 25, 27, 30, 33, 34, 39, 40, 45, 47, 50, 51]). Depiction of the full extracted data on the relationships between the individual MRI measures and each cognitive test are available in Supplementary Tables 1, 2, and 3 as an online appendix (see Supplementary Material available online at http://dx.doi.org/10.1155/2014/975803). In studies evaluating T2LV and PASAT, half of the studies evaluated RRMS patients and the remaining half evaluated mostly mixed MS populations with a small number of studies identified as benign MS or clinically isolated syndrome (CIS) patients. Similar proportions of MS subtypes were observed across studies reporting correlations between T2LV and SDMT as half of the studies evaluated mixed-disease-course patients and the remaining studies evaluated homogeneous populations on relapsing-remitting MS (RRMS), benign MS, or probable MS. Studies tended to report only significant correlations between IPS measures and MRI outcomes, suggesting reporting bias. Data were sufficient to conduct meta-analyses on pure RRMS populations and studies evaluating a mix of MS subtypes. A pooled meta-analysis of all studies was not conducted. However, the Holm-Bonferroni procedure was used to conduct significance testing on the relationship between MRI measures and IPS across all studies [16].

### 3.1. SDMT and MRI Measures

There was a consistent relationship between the SDMT and whole-brain MRI measures, a relationship that was strongest in mixed MS populations. Eighteen studies meeting criteria to analyze the relationship between SDMT and T2LV and 18 studies for SDMT and brain atrophy were identified, though six studies from each comparison did not report correlations.

**Figure 2:**
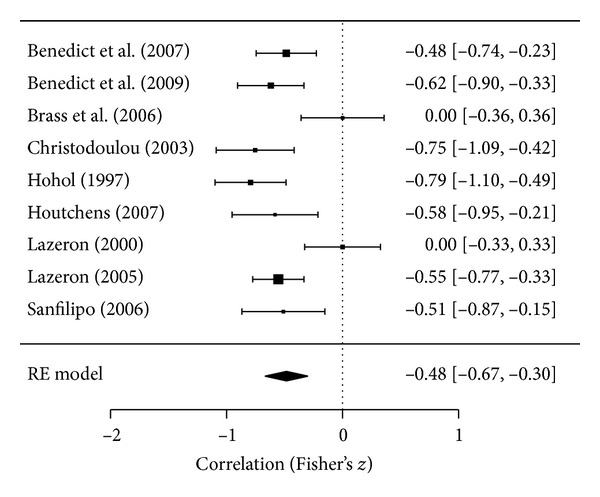
Correlation between T2LV and SDMT processing speed in patients with mixed MS subtypes.

In studies evaluating RRMS patients, there was a significant relationship between SDMT and T2LV, with reported correlations ranging from weak (*r* = −0.22) to strong (*r* = −0.51). Five [13, 24, 30, 45, 50] of the seven [13, 18, 24, 30, 45, 47, 50] studies (71.4%) assessing RRMS patients reported significant correlations. In patients with a mix of MS subtypes, a moderate-to-strong correlation was observed between SDMT and T2LV as *r*-values ranged from −0.45 to −0.89. Seven [20, 22, 27, 33, 34, 39, 51] of nine [20, 22, 25, 27, 33, 34, 39, 40, 51] studies (77.7%) assessing patients with mixed MS subtypes reported correlations between SDMT and T2LV, six of which were significant [20, 22, 27, 33, 34, 39] and one in which the significance was not reported [51]. These seven studies were eligible for meta-analysis due to the reporting of near-complete data. In meta-analyzing the relationship between SDMT and T2LV in mixed MS patients, zeros were imputed for two studies [25, 40] that did not report correlations, resulting in an estimate of *r* = −0.45, *P* < 0.001; meta-analysis results are depicted in Figure 2. Standard tests of statistical heterogeneity and for publication bias were not applicable due to the imputations.

**Figure 3:**
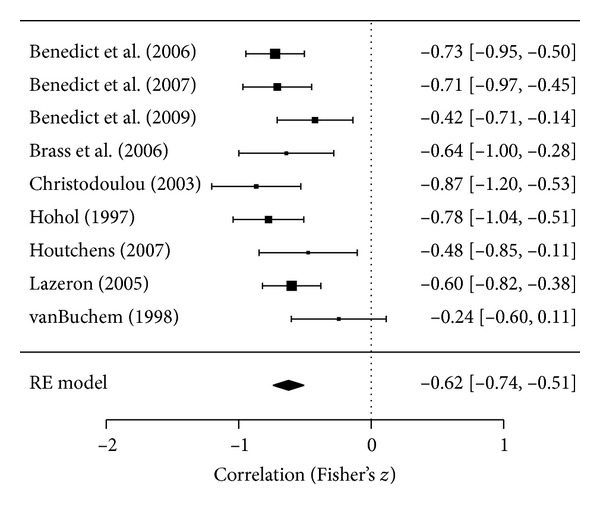
Correlation between brain atrophy and SDMT processing speed in patients  with mixed MS subtypes.

Studies evaluating atrophy and SDMT found a moderate-to-strong correlation between these two variables as *r*-values ranged from −0.40 to −0.73, indicating that greater atrophy was associated with poorer SDMT performance. All 10 studies [20–23, 25, 27, 33, 34, 39, 54] assessing patients with mixed MS subtypes reported correlations, eight of which were significant [20–23, 27, 33, 34, 39] and one [25] in which the statistical significance was not reported. In studies on RRMS patients, only two [21, 24] of seven [13, 18, 21, 24, 30, 47, 48] studies reported significant correlation between brain atrophy and SDMT. The nine studies [20–22, 25, 27, 33, 34, 39, 54] reporting correlations in the patients with mixed MS subtypes were meta-analyzable, and one study (which reported a significant effect) could not be included due to the nature of the reported effect [23]. A direct meta-analysis of the correlations in the nine studies found a strong mean correlation between SDMT and brain atrophy in patients with mixed MS subtypes (*r* = −0.54, *P* < 0.001) and there was no sign of statistical heterogeneity (*P* = 0.18) or publication bias (*P* = 0.30), demonstrating that the correlations between atrophy and SDMT were consistent across the nine papers examining these outcomes. Meta-analysis results for this correlation are depicted in Figure 3.

### 3.2. PASAT and MRI Measures

There was a consistent relationship between the PASAT and whole-brain MRI measures, which was strongest between PASAT and brain atrophy. Twenty-two studies (with 23 significance tests) that met the criteria to analyze the relationship between PASAT and T2LV and 24 studies for PASAT and brain atrophy were identified, though 10 and 11 studies did not report significant correlations, respectively.

In studies evaluating RRMS patients, the relationship reported between PASAT and T2LV varied from weak to strong, with *r*-values ranging from −0.10 to −0.40. However, over half of studies (53.8%) [13, 18, 38, 42, 44, 47, 48] did not report correlations in RRMS patients, despite measuring T2LV and administering the PASAT test. Studies that evaluated MS patients with mixed disease courses found that correlations varied between T2LV and the PASAT test, but the relationship was strong in most studies (weak −0.23 to strong −0.58) reporting significant results. Nine [20, 25, 27, 28, 31, 33, 34, 36, 39, 52] of the 12 studies [20, 25, 27, 28, 31, 33, 34, 36, 39, 40, 52] (75%) assessing patients with a mix of MS subtypes reported significant correlations.

In RRMS patients, a moderate correlation was reported between atrophy and the PASAT test in half of studies (*r*-values ranged from −0.30 to −0.40); the remaining half of studies (*n* = 5) did not report significant results. In populations with mixed MS subtypes, correlations between atrophy and the PASAT were consistently strong, with *r*-values ranging from −0.43 to −0.59. Seven [20, 23, 27, 33, 34, 39, 54] of the 11 [20, 23, 25, 27, 29, 31, 33, 34, 39, 49, 54] studies (63.6%) assessing patients with a mix of MS subtypes reported significant correlations. Meta-analyses on relationships between PASAT and the MRI measures were not possible due to a high proportion of missing data in studies. However, the Holm-Bonferroni method was used to conduct familywise testing. The results of this test suggest confirmed relationships for four of the six studies reporting significant relationships between PASAT and T2LV. The results of the test can be found in Table 1.

### 3.3. Atrophy and SDMT versus Atrophy and PASAT

The correlation between atrophy and SDMT was stronger than that between atrophy and PASAT. Seventeen studies evaluated the relationship between T2LV and the SDMT and PASAT cognitive tests. The relationship was strongest in populations with mixed MS subtypes. In mixed MS patients, the magnitude of the correlations between brain atrophy and PASAT ranged from *r* = −0.24 to −0.67, and correlations between brain atrophy and SDMT ranged from *r* = −0.40 to −0.73. Significant results were reported in all seven studies [20, 25, 29, 33, 34, 39, 54] evaluating patients with a mix of MS subtypes. In RRMS patients, only two [24, 33] of seven [13, 18, 24, 30, 33, 47, 48] studies reported significant correlations. A longitudinal study conducted in RRMS patients found a strong correlation between the change in brain volume and change in PASAT score over one year (*r* = 0.64) and an even stronger correlation between the change in brain volume and change in SDMT score over the same period (*r* = 0.75) [33]. In the second study, a significant correlation was found between atrophy and SDMT or PASAT (*r* > 0.4 for both) in only patients with high educational levels (those with at least 12 years of education) [24].

### 3.4. T2LV and SDMT versus T2LV and PASAT

There was a stronger correlation between T2LV and the SDMT than T2LV and the PASAT in both RRMS patients and studies with a mix of MS subtypes. Seventeen studies evaluated T2LV, SDMT, and PASAT, but only 52% reported values for correlations between the MRI measure and both cognitive tests. In patients with mixed MS subtypes, the magnitude of the correlations between T2LV and PASAT ranged from *r* = −0.23 to −0.58, and correlations between T2LV and SDMT ranged from *r* = −0.45 to −0.66. Significant results were reported in 57.1% (four out of seven) of studies evaluating patients with mixed MS subtypes. In RRMS patients, only four of seven [24, 30, 45, 50] studies (71.4%) reported significant correlations, which ranged from −0.10 to −0.34 between T2LV and PASAT, and four of seven studies (57.1%) reported significant correlations ranging from −0.22 to −0.51 between T2LV and SDMT.

### 3.5. Comparisons between the PASAT 2- and 3-Second Tests

There was no apparent trend showing that the results for either the PASAT 2-second or the 3-second test were more strongly correlated with T2LV or brain atrophy. Nine studies reported correlations between T2LV and both the PASAT 2- and 3-second tests [18, 24, 27, 30, 39, 45, 47–49] and seven studies reported correlations between T2LV and both the PASAT 2- and 3-second tests [27, 30, 39, 47–49]. In studies that reported significant results for both tests, similar correlations were observed.

### 3.6. Comparisons between Brain Atrophy and T2LV

There was no evidence that either T2LV or atrophy was more strongly correlated with PASAT score. Similarly, there was no evidence that either T2LV or atrophy was more strongly correlated with SDMT results. The *P* value for binomial tests conducted to determine whether one set of correlations was stronger than the other (T2LV and PASAT versus atrophy and PASAT) that was 0.72 and (T2LV and SDMT versus atrophy and SDMT) and 0.13 for the respective comparisons, demonstrating that there was no evidence of a trend in favor of one MRI measure over the other.

## 4. Discussion

This is the first systematic review conducted to date on studies assessing the relationship between whole-brain conventional MRI measures and IPS dysfunction in MS. Several conclusions can be drawn from this meta-analysis. First, moderate-to-strong correlations exist between impaired conventional MRI measures of lesion volume and atrophy and psychometric performance on IPS measures in populations with a mix of MS subtypes. Second, evidence of a relationship in RRMS-only patients is sparse. Third, correlations with both MRI measures were stronger for the SDMT than for the PASAT. Finally, correlations between IPS measures and T2LV or atrophy were of roughly equal. These findings do not appear to be the result of study and population heterogeneity or reporting bias.

These results provide additional validation for replacing the PASAT with the SDMT as the sole measure of cognition in the MSFC. Our review indicates that the SDMT is superior to the PASAT in correlating with underlying brain pathology as measured by conventional MR measures. Not surprisingly, the SDMT was recently selected as the sole measure of cognition to be included in all studies funded by the National Institute of Neurological Disorders and Stroke [55].

A surprising result is the lack of evidence that T2LV and whole-brain atrophy have different sensitivities to IPS dysfunction. Several investigators (e.g., Benedict et al., 2004) [56] have suggested that atrophy provides a better indicator of cognitive performance than white matter lesion volume. Our review does not support this hypothesis. It is important to note that our review emphasized whole-brain atrophy and IPS measures. It is conceivable that if we included regional atrophy measures or other cognitive functions (e.g., episodic memory), our results may be different.

When evaluating correlations by disease state, moderate-to-strong relationships were consistently reported in patients with mixed MS subtypes compared to RRMS patients in studies evaluating atrophy and SDMT or PASAT as well as T2LV and SDMT or PASAT. It is possible that this is in part a “restriction-of-range” issue with regard to disease severity and cognitive function. Patients in mixed-MS studies will generally have a greater range of both disease severity and cognitive ability, which will make it easier to detect relationships between the two. While there was a great deal of missing data on these two factors, there was evidence to suggest that patients in mixed-MS studies had a higher level of cognitive decline.

There was a paucity of data reported on the CIS, SPMS, and PPMS populations as most studies identified in this review evaluated a purely RRMS population or a mixed-MS disease course population. In the few studies identified on these MS subtypes, a clear relationship could not be determined between MRI measures and information processing performance, as measured by the SDMT and PASAT. There may be factors associated with these less common subtypes that affect the relationship between these variables, as in many cases we observed stronger correlations between MRI characteristics and cognitive status among patients with a mix of MS subtypes compared with RRMS patients. The differences in disease duration and disability status also may affect the relationship between these variables as patients with more advanced disease may experience a greater degree of cognitive impairment.

In general, study populations were somewhat heterogeneous in respect to both the patient populations and disease measurements. Studies differed in the specific way atrophy was measured and controlled for different variables; however, when capturing data we did not use correlations for which endogenous variables, such as depression, were controlled for. Measures of atrophy were diverse, while the proportion of patients with cognitive impairment was not consistently reported in studies. This heterogeneity impacts the generalizability of meta-analysis results. If a more homogenous population were available, results may differ.

Several methodological issues should be highlighted. First, the high prevalence of reporting bias among the studies limits the number of analyses that were possible to assess the strength of correlations between MRI and cognitive measures. Many studies investigated more than a dozen cognitive outcomes and/or MRI outcomes, and numeric estimates of strength were only reported for those with significant results. Thus, we were often not able to estimate the strength of a relationship; however, by adopting the conservative assumption that any given unreported relationship was not significant due to reporting bias, we were able to test global null hypotheses through the Holm-Bonferroni procedure.

Tied to the reporting bias is the fact that many of the smaller studies (e.g., *n* < 50) had low power to detect significant effects. The smaller the sample size, the greater the chance that unless a given relationship had a high correlation (*r* ≥ 0.40), it would be unreported, especially if the authors had many different MRI and cognition measures to discuss.

As noted, the high proportion of missing data on correlations (ranging from 33% for SDMT/T2LV to 46% for atrophy/PASAT) precluded a robust numeric estimation of mean correlations between all MRI measures and cognitive measures. Meta-analyses were only possible on the relationship between SDMT and T2LV and SDMT and brain atrophy.

We also note that some studies reported only correlations for an overall battery of measures, such as the Brief Repeatable Battery, where results were only reported as a composite score rather than correlations for individual tests. In these cases, the assessment of the relationship between cognitive measures and SDMT or PASAT was prohibited. Finally, exploring tests measuring performance for the other cognitive domains may yield different results regarding the strength of correlations as this review focused on SDMT and PASAT.

## 5. Conclusions and Clinical Implications

This systematic review and meta-analysis provides additional justification for replacing the PASAT with the SDMT as the sole measure of cognition in the MSFC. The finding of equivalent correlations of IPS measures with T2LV and brain atrophy has clinical implications. Severity of atrophy is often difficult to perceive without quantitative assessment and statistical correction for age. In contrast, the severity of T2LV can be readily appreciated by an experienced MS clinician. High white matter lesion load, therefore, would increase the suspicion that the patient is experiencing IPS dysfunction and could prompt a referral for neuropsychological assessment.

## Supplementary Material

Supplementary tables 1, 2, and 3 depict the raw data abstracted from the included studies identified by the review. Each row represents a study with any reported sub-groups separated by a solid line as a nested row. Study References are listed using a truncated format in the first column and full citations are available in the reference list of the manuscript. Separate columns report the available information on disease course, study sample size, the proportion of patients with cognitive impairment, and the specific cognitive test evaluated. The correlations between measures with the reported statistical significance and mean score are also depicted. For studies evaluating the relationship between the information processing measures and brain atrophy, a column reporting the approach used to measure brain volume is indicated in a separate column.

## Figures and Tables

**Table 1:** Holm-Bonferroni Investigation into the relationships between whole-brain MRI measures and information processing tests.

MRI measure	Cognitive measure	Number of tests	Population	Number of null hypotheses rejected	Smallest *P* value	Threshold	Number of NS results (*P* > 0.05)
T2LV	SDMT	18	All	8	<0.0001	0.0028	7
T2LV	SDMT	7	RRMS only	3	<0.0001	0.0073	2
T2LV	SDMT	9	Mixed only	6	<0.001	0.0057	3
T2LV	PASAT	27	All	4	<0.001	0.0019	15
T2LV	PASAT	13	RRMS only	0	<0.01	0.0039	8
T2LV	PASAT	12	Mixed only	4	<0.001	0.0043	6
Atrophy	SDMT	20	All	6	<0.001	0.0026	8
Atrophy	SDMT	7	RRMS only	1	<0.01	0.0073	5
Atrophy	SDMT	11	Mixed only	9	<0.001	0.0047	2
Atrophy	PASAT	23	All	4	<0.0001	0.0022	13
Atrophy	PASAT	10	RRMS only	0	0.045	0.0051	7
Atrophy	PASAT	11	Mixed only	4	<0.0001	0.0047	6

The most significant *P* value in an analysis had to be lower than the threshold in order to reject any null hypotheses. Reported *P* values are assumed equal to the maximum possible, for example, *P* < 0.01 is tested as *P* = 0.01. Where multiple *P* values are reported for the same relationship (possibly adjusted versus unadjusted), the most insignificant *P* value was used. Number of studies with RRMS + Mixed only do not necessarily sum to total studies, as some studies had a 100% SPMS or benign MS population. “Number of tests” strongly corresponds to number of studies; rarely, studies had data on subgroups that could not be combined.

MRI: magnetic resonance imaging; PASAT: paced auditory serial addition test; RRMS: relapse-remitting multiple sclerosis; SDMT: symbol digit modalities test; T2LV: T2-weighted lesion volume; NS: not significant.

## References

[1] Bobholz JA, Rao SM (2003). Cognitive dysfunction in multiple sclerosis: a review of recent developments. *Current Opinion in Neurology*.

[2] Heaton RK, Nelson LM, Thompson DS, Burks JS, Franklin GM (1985). Neuropsychological findings in relapsing-remitting and chronic-progressive multiple sclerosis. *Journal of Consulting and Clinical Psychology*.

[3] Rao SM, Leo GJ, Bernardin L, Unverzagt F (1991). Cognitive dysfunction in multiple sclerosis. I. Frequency, patterns, and prediction. *Neurology*.

[4] Gronwall DMA (1977). Paced auditory serial addition task: a measure of recovery from concussion. *Perceptual and Motor Skills*.

[5] Cutter GR, Baier ML, Rudick RA (1999). Development of a multiple sclerosis functional composite as a clinical trial outcome measure. *Brain*.

[6] Fischer JS, Rudick RA, Cutter GR, Reingold SC (1999). The multiple sclerosis functional composite measure (MSFC): an integrated approach to MS clinical outcome assessment. National MS Society Clinical Outcomes Assessment Task Force. *Multiple Sclerosis*.

[7] Rudick R, Antel J, Confavreux C (1996). Clinical outcomes assessment in multiple sclerosis. *Annals of Neurology*.

[8] Rudick R, Antel J, Confavreux C (1997). Recommendations from the national multiple sclerosis society clinical outcomes assessment task force. *Annals of Neurology*.

[9] Rao SM (1990). *A Manual for the Brief Repeatable Battery of Neuropsychological Tests in Multiple Sclerosis*.

[10] Benedict RHB, Fischer JS, Archibald CJ (2002). Minimal neuropsychological assessment of MS patients: a consensus approach. *Clinical Neuropsychologist*.

[11] Langdon DW, Amato MP, Boringa J (2012). Recommendations for a brief international cognitive assessment for multiple sclerosis (BICAMS). *Multiple Sclerosis*.

[12] Fisk JD, Archibald CJ (2001). Limitations of the Paced Auditory Serial Addition Test as a measure of working memory in patients with multiple sclerosis. *Journal of the International Neuropsychological Society*.

[13] Brochet B, Deloire MSA, Bonnet M (2008). Should SDMT substitute for PASAT in MSFC? A 5-year longitudinal study. *Multiple Sclerosis*.

[14] Drake AS, Weinstock-Guttman B, Morrow SA, Hojnacki D, Munschauer FE, Benedict RHB (2010). Psychometrics and normative data for the multiple sclerosis functional composite: replacing the PASAT with the symbol digit modalities test. *Multiple Sclerosis*.

[15] Filippi M, Rocca MA, Benedict RHB (2010). The contribution of MRI in assessing cognitive impairment in multiple sclerosiss. *Neurology*.

[16] Holm S (1979). A simple sequentially rejective multiple test procedure. *Scandinavian Journal of Statistics*.

[17] Achiron A, Barak Y (2003). Cognitive impairment in probable multiple sclerosis. *Journal of Neurology, Neurosurgery and Psychiatry*.

[18] Amato MP, Portaccio E, Goretti B (2010). Relevance of cognitive deterioration in early relapsing-remitting MS: a 3-year follow-up study. *Multiple Sclerosis*.

[19] Amato MP, Portaccio E, Stromillo ML (2008). Cognitive assessment and quantitative magnetic resonance metrics can help to identify benign multiple sclerosis. *Neurology*.

[20] Benedict RHB, Bruce J, Dwyer MG (2007). Diffusion-weighted imaging predicts cognitive impairment in multiple sclerosis. *Multiple Sclerosis*.

[21] Benedict RHB, Bruce JM, Dwyer MG (2006). Neocortical atrophy, third ventricular width, and cognitive dysfunction in multiple sclerosis. *Archives of Neurology*.

[22] Benedict RHB, Ramasamy D, Munschauer F, Weinstock-Guttman B, Zivadinov R (2009). Memory impairment in multiple sclerosis: correlation with deep grey matter and mesial temporal atrophy. *Journal of Neurology, Neurosurgery and Psychiatry*.

[23] Benedict RHB, Zivadinov R, Carone DA (2005). Regional lobar atrophy predicts memory impairment in multiple sclerosis. *American Journal of Neuroradiology*.

[24] Bonnet MC, Deloire MSA, Salort E, Dousset V, Petry KG, Brochet B (2006). Evidence of cognitive compensation associated with educational level in early relapsing-remitting multiple sclerosis. *Journal of the Neurological Sciences*.

[25] Brass SD, Benedict RHB, Weinstock-Guttman B, Munschauer F, Bakshi R (2006). Cognitive impairment is associated with subcortical magnetic resonance imaging grey matter T2 hypointensity in multiple sclerosis. *Multiple Sclerosis*.

[26] Camp SJ, Stevenson VL, Thompson AJ (2005). A longitudinal study of cognition in primary progressive multiple sclerosis. *Brain*.

[27] Christodoulou C, Krupp LB, Liang Z (2003). Cognitive performance and MR markers of cerebral injury in cognitively impaired MS patients. *Neurology*.

[28] Ciccarelli O, Brex PA, Thompson AJ, Miller DH (2002). Disability and lesion load in MS: a reassessment with MS functional composite score and 3D fast FLAIR. *Journal of Neurology*.

[29] Cortese I, Ohayon J, Fenton K, Griffith H, Reich DS, Bielekova B Comparison of SDMT and PASAT in multiple sclerosis.

[30] Deloire MSA, Salort E, Bonnet M (2005). Cognitive impairment as marker of diffuse brain abnormalities in early relapsing remitting multiple sclerosis. *Journal of Neurology, Neurosurgery and Psychiatry*.

[31] Edwards SGM, Liu C, Blumhardt LD (2001). Cognitive correlates of supratentorial atrophy on MRI in multiple sclerosis. *Acta Neurologica Scandinavica*.

[32] Hildebrandt H, Hahn HK, Kraus JA, Schulte-Herbrüggen A, Schwarze B, Schwendemann G (2006). Memory performance in multiple sclerosis patients correlates with central brain atrophy. *Multiple Sclerosis*.

[33] Hohol MJ, Guttmann CRG, Orav J (1997). Serial neuropsychological assessment and magnetic resonance imaging analysis in multiple sclerosis. *Archives of Neurology*.

[34] Houtchens MK, Benedict RHB, Killiany R (2007). Thalamic atrophy and cognition in multiple sclerosis. *Neurology*.

[35] Ingle GT, Stevenson VL, Miller DH, Thompson AJ (2003). Primary progressive multiple sclerosis: a 5-year clinical and MR study. *Brain*.

[36] Kalkers NF, Bergers L, de Groot V (2001). Concurrent validity of the MS Functional Composite using MRI as a biological disease marker. *Neurology*.

[37] Khalil M, Enzinger C, Langkammer C (2011). Cognitive impairment in relation to MRI metrics in patients with clinically isolated syndrome. *Multiple Sclerosis*.

[38] Lanzillo R, Prinster A, Scarano V (2006). Neuropsychological assessment, quantitative MRI and ApoE gene polymorphisms in a series of MS patients treated with IFN beta-1b. *Journal of the Neurological Sciences*.

[39] Lazeron RHC, Boringa JB, Schouten M (2005). Brain atrophy and lesion load as explaining parameters for cognitive impairment in multiple sclerosis. *Multiple Sclerosis*.

[40] Lazeron RHC, Langdon DW, Filippi M (2000). Neuropsychological impairment in multiple sclerosis patients: the role of (juxta)cortical lesion on FLAIR. *Multiple Sclerosis*.

[41] Lin X, Tench CR, Morgan PS, Constantinescu CS (2008). Use of combined conventional and quantitative MRI to quantify pathology related to cognitive impairment in multiple sclerosis. *Journal of Neurology, Neurosurgery and Psychiatry*.

[42] Locatelli L, Zivadinov R, Grop A, Zorzon M (2004). Frontal parenchymal atrophy measures in multiple sclerosis. *Multiple Sclerosis*.

[43] Mesaros S, Rocca MA, Riccitelli G (2009). Corpus callosum damage and cognitive dysfunction in benign MS. *Human Brain Mapping*.

[44] Mineev KK, Prakhova LN, Il’ves AG (2009). Characteristics of neurological and cognitive status in patients with multiple sclerosis in relation to the location and volumes of demyelination foci and the severity of brain atrophy. *Neuroscience and Behavioral Physiology*.

[45] Patti F, Amato MP, Trojano M (2009). Cognitive impairment and its relation with disease measures in mildly disabled patients with relapsing-remitting multiple sclerosis: baseline results from the Cognitive Impairment in Multiple Sclerosis (COGIMUS) study. *Multiple Sclerosis*.

[46] Penny S, Khaleeli Z, Cipolotti L, Thompson A, Ron M (2010). Early imaging predicts later cognitive impairment in primary progressive multiple sclerosis. *Neurology*.

[47] Portaccio E, Amato MP, Bartolozzi ML (2006). Neocortical volume decrease in relapsing-remitting multiple sclerosis with mild cognitive impairment. *Journal of the Neurological Sciences*.

[48] Portaccio E, Goretti B, Zipoli V (2009). APOE-*ε*4 is not associated with cognitive impairment in relapsing-remitting multiple sclerosis. *Multiple Sclerosis*.

[49] Rao SM, Leo GJ, Haughton VM, St Aubtin-Faubert P, Bernardin L (1989). Correlation of magnetic resonance imaging with neuropsychological testing in multiple sclerosis. *Neurology*.

[50] Rovaris M, Iannucci G, Falautano M (2002). Cognitive dysfunction in patients with mildly disabling relapsing-remitting multiple sclerosis: an exploratory study with diffusion tensor MR imaging. *Journal of the Neurological Sciences*.

[51] Sanfilipo MP, Benedict RHB, Weinstock-Guttman B, Bakshi R (2006). Gray and white matter brain atrophy and neuropsychological impairment in multiple sclerosis. *Neurology*.

[52] Snyder PJ, Cappelleri JC (2001). Information processing speed deficits may be better correlated with the extent of white matter sclerotic lesions in multiple sclerosis than previously suspected. *Brain and Cognition*.

[53] Sumowski JF, Chiaravalloti N, Wylie G, Deluca J (2009). Cognitive reserve moderates the negative effect of brain atrophy on cognitive efficiency in multiple sclerosis. *Journal of the International Neuropsychological Society*.

[54] van Buchem MA, Grossman RI, Armstrong C (1998). Correlation of volumetric magnetization transfer imaging with clinical data in MS. *Neurology*.

[55] Common Data Elements Multiple Sclerosis. http://www.commondataelements.ninds.nih.gov/ms.aspx#tab=Data_Standards.

[56] Benedict RHB, Carone DA, Bakshi R (2004). Correlating brain atrophy with cognitive dysfunction, mood disturbances, and personality disorder in multiple sclerosis. *Journal of Neuroimaging*.

