# Supplementary material for: Correlations between MRI and Information Processing Speed in MS: A Meta-Analysis
Source: Mult Scler Int. 2014 Mar 25;2014:975803. doi: 10.1155/2014/975803 (PMC3984845; doi:10.1155/2014/975803)
Supplement: Supplementary file 1 — Supplementary tables 1, 2, and 3 depict the raw data abstracted from the included studies identified by the review. Each row represents a study with any reported sub-groups separated by a solid line as a nested row. Study References are listed using a truncated format in the first column and full citations are available in the reference list of the manuscript. Separate columns report the available information on disease course, study sample size, the proportion of patients with cognitive impairment, and the specific cognitive test evaluated. The correlations between measures with the reported statistical significance and mean score are also depicted. For studies evaluating the relationship between the information processing measures and brain atrophy, a column reporting the approach used to measure brain volume is indicated in a separate column. [file 975803.f1.pdf]

TITLE: Correlations between MRI and Information Processing Speed in MS: A Meta-Analysis

JOURNAL: Multiple Sclerosis Journal

AUTHORS: S.M. Rao, PhD[1]; A. Martin, BS[2]; R. Huelin, BA[2]; E. Wissinger, PhD[2]; Z. Khankhel, BS[2]; E. Kim, MD[3]; K. Fahrbach, PhD[2]

CORRESPONDING AUTHOR: Amber Martin, Evidera, amber.martin@evidera.com

Table 1: Supplementary Table 1 **MS Studies Evaluating Correlations between T2LV and SDMT and/or T2LV and PASAT**

Reference	Disease Course	Cognitive Test	Sample Size	r-value	p-value	Mean (SD) Score	Cognitively Impaired
Amato 2008	Benign MS	SDMT	47	NS		NR	23.4%
Achiron 2003	Probable MS	SDMT	67	NR	NR	NR	NR
Brass 2006	Mixed	SDMT	33	NS		NR	NR
Benedict 2007	Mixed	SDMT	60	-0.45	<0.01	NR	61.7%
Benedict 2009	Mixed	SDMT	50	-0.55	<0.001	NR	NR
Christodoulou 2003	Mixed	SDMT	37	-0.637	0.01	47.0 (14.1)	100%*
Hohol 1997	Mixed: CPMS, SPMS, anc PPMS	SDMT	13	-0.89	<0.001	NR	NR
	Mixed: RRMS, CPMS, SPI PPMS	SDMT	44	-0.66	<0.001	41.5 (11.6)	NR
Houtchens 2007	Mixed	SDMT	31	-0.524	<0.001	48.2 (19)	NR
Lazeron 2000	Mixed	SDMT	39	NS		44.9 (14.4)	21%
Lazeron 2005	Mixed	SDMT	82	-0.503	<0.001	45.22 (13.67)	67%
Sanfilipo 2006	Mixed	SDMT	33	-0.47	NR	45.0 (10.7)	NR
Amato 2010	RRMS	SDMT	49	NS		48.7 (13.3)	30.6%
Bonnet 2006	RRMS, Low education gr	SDMT	19	<0.30	NR	36.31 (8.49)	NR
	RRMS, High education gı	SDMT	24	>0.40	<0.05	46.33 (11.46)	NR

Reference	Disease Course	Cognitive Test	Sample Size	r-value	p-value	Mean (SD) Score	Cognitively Impaired
Brochet 2008	RRMS	SDMT	46	-0.31	0.032	NR	NR
Deloire 2005	RRMS	SDMT	58	-0.51	<0.0001	41.19 (12.49)	45%
Patti 2009	RRMS	SDMT	327	-0.219	<0.001	NR	22%
Portaccio 2006	RRMS	SDMT	41	NS		NR	56%
Rovaris 2002	RRMS	SDMT	34	-0.49	0.003	NR	26.5%
Achiron 2003	Probable MS	PASAT-3	67	NS		NR	NR
Amato 2008	Benign MS	PASAT	47	NR	NR	NR	23.4%
Mesaros 2009	Benign MS	PASAT-3	54	-0.32	0.03	NR	17%
Amato 2010	RRMS	PASAT-2	49	NS		28.3 (9.9)	30.6%
		PASAT-3	49	NS		39.7 (12.4)	30.6%
Bonnet 2006	RRMS - Low educational level	PASAT-2	19	<0.30	NR	25.1 (16.26)	NR
		PASAT-3	19	<0.30	NR	36.31 (16.46)	NR
		PASAT-2	24	<0.30	NR	37.62 (8.98)	NR
		PASAT-3	24	>0.40	<0.05	47.41 (8.63)	NR
Brochet 2008	RRMS	PASAT	46	NS		NR	NR
Deloire 2005	RRMS	PASAT-2	58	NR	NR	32.70 (13.35)	45%
		PASAT-3	58	-0.34	<0.05	42.43 (13.57)	45%
Lanzillo 2006	RRMS	PASAT	52	NR	<0.01	NR	46%
Lin 2008	RRMS	PASAT-3	36	-0.4	0.017	NR	25%

Reference	Disease Course	Cognitive Test	Sample Size	r-value	p-value	Mean (SD) Score	Cognitively Impaired
Locatelli 2004	RRMS	PASAT	39	NR	NR	44.9 (14.9)	NR
Mineev 2009	RRMS	PASAT-3	65	NR	NR	NR	NR
Patti 2009	RRMS	PASAT-3	327	-0.113	0.057		22%
		PASAT-2	327	-0.103	0.083		22%
Portaccio 2006	RRMS	PASAT-2	41	NS		NR	56%
		PASAT-3	41	NS		NR	56%
Portaccio 2009	RRMS	PASAT-2	50	NR	NR	NR	33%
		PASAT-3	50	NR	NR	NR	33%
Rovaris 2002	RRMS	PASAT	34	<0.30	NR	NR	26.5%
Edwards 2001	Mixed	PASAT	40	0.51	0.001	NR	NR
Hohol 1997	SPMS + PPMS	PASAT-3	13	-0.26	0.47	NR	NR
	Mixed	PASAT-3	44	-0.58	<0.001	69.7 (21.3)	NR
Houtchens 2007	Mixed	mean of PASAT-2 and -3	31	-0.501	0.002	35.1 (14)	NR
Benedict 2007	Mixed	PASAT	60	-0.23	>0.05	NR	61.7%
Brass 2006	Mixed	PASAT	33	NS		NR	NR
Christodoulou 2003	Mixed	PASAT-3	37	-0.504	=0.01	39.3 (13.6)	100%^
		PASAT-2	37	-0.309	=0.1	29.1 (10.1)	100%^
Ciccarelli 2002	Mixed (conventional	PASAT-3	39	-0.29	>0.05	46 (14-60)*	NR

Reference	Disease Course	Cognitive Test	Sample Size	r-value	p-value	Mean (SD) Score	Cognitively Impaired
	T2LV)						
	Mixed (T2LV using FLAIR)	PASAT-3	39	-0.26	>0.05	46 (14-60)*	NR
Kalkers 2001	PPMS	PASAT-3	29	NS		45 (22-52)	NR
	SPMS	PASAT-3	41	-0.44	=0.01	37 (30-49)*	NR
	RRMS	PASAT-3	64	-0.28	=0.05	48 (39-55)*	NR
	SPMS + PPMS	PASAT-3	70	-0.36	>0.05	NR	NR
	Mixed	PASAT-3	134	-0.27	=0.01	46 (32-52)*	NR
Lazeron 2000	Mixed	mean of PASAT-2 and -3	39	NS		72.3 (27.2)	21%
Lazeron 2005	Mixed	PASAT-3	82	-0.405	<0.001	39.7 (18.93)	67%
		PASAT-2	82	-0.374	<0.001	29.44 (15.36)	67%
Rao 1989	Mixed	PASAT-2	53	NS		NR	36%
		PASAT-3	53	NS		NR	36%
Snyder 2001	Mixed	PASAT - dyad	-0.40	0.01	41	10.15 (9.46)	NR

* Median (range)

^ Mild cognitive impairment

CPMS = Chronic progressive multiple sclerosis; MS = Multiple sclerosis; NR = Not reported; NS = Non-significant; PASAT = Paced auditory serial addition test; PPMS = Primary progressive multiple sclerosis; RRMS = Relapse-remitting multiple sclerosis; SD = Standard deviation; SPMS = Secondary progressive multiple sclerosis; T2LV = T2-weighted lesion volume

Table 2: Supplementary Table 2 **MS Studies Evaluating Correlations between Brain Atrophy and SDMT and/or Atrophy and PASAT**

Reference	Disease Course	Cognitive Test	MRI Measure	Sample Size	*r*-value	p-value	Mean (SD) Score	Cognitively Impaired
Amato 2008	Benign MS	SDMT	Atrophy (NBV)	47	NR	NR	NR	23.4%
Benedict 2005	Mixed	SDMT	Atrophy (Mixed)	31	0.63	<0.001	NR	NR
Benedict 2006	Mixed	SDMT	Atrophy (TVW)	82	-0.62	<0.001	NR	59.8%
	RRMS	SDMT	Atrophy (TVW)	NR	-0.44	<0.005	NR	NR
	SPMS	SDMT	Atrophy (TVW)	NR	-0.62	<0.005	NR	NR
Benedict 2007	Mixed	SDMT	Atrophy (BPF)	60	0.61	<0.01	NR	61.7%
Benedict 2009	Mixed	SDMT	Atrophy (NBV)	50	0.4	<0.01	NR	NR
Brass 2006	Mixed	SDMT	(BPF)	33	0.57	NR	NR	NR
Christodoulou 2003	Mixed	SDMT	Atrophy (Central)	37	-0.7	0.01	47.0 (14.1)	100%*
Hohol 1997	Mixed: CPMS, SPMS, and	SDMT	Atrophy (BICVR)	13	0.73	0.02	NR	NR

Reference	Disease Course	Cognitive Test	MRI Measure	Sample Size	*r*-value	p-value	Mean (SD) Score	Cognitively Impaired
	PPMS							
	Mixed: RRMS, CPMS, SPMS, PPMS	SDMT	Atrophy (BICVR)	44	0.63	<0.001	41.5 (11.6)	NR
Houtchens 2007	Mixed	SDMT	Atrophy (TVW)	31	-0.44	0.007	48.2 (19)	NR
		SDMT	Atrophy (BPF)	31	0.57	<0.001	48.2 (19)	NR
Lazeron 2005	Mixed	SDMT	Atrophy (RBV)	82	0.54	<0.001	45.22 (13.67)	67%
van Buchem 1998	Mixed	SDMT	Atrophy (BV)	33	0.24	0.14	NR	54%
Cortese 2011	NR	SDMT	Atrophy (SBV)	51	0.39	0.006	NR	NR
Amato 2010	RRMS	SDMT	Atrophy (PBVC)	41	NS		NR	43.9%

Reference	Disease Course	Cognitive Test	MRI Measure	Sample Size	*r*-value	p-value	Mean (SD) Score	Cognitively Impaired
Bonnet 2006	RRMS, Low education group	SDMT	Atrophy (VF)	19	<0.30	NR	36.31 (8.49)	NR
	RRMS, Low education group	SDMT	Atrophy (BPF)	19	<0.30	NR	36.31 (8.49)	NR
	RRMS, High education group	SDMT	Atrophy (VF)	24	<0.30	NR	46.33 (11.46)	NR
	RRMS, High education group	SDMT	Atrophy (BPF)	24	>0.40	<0.05	46.33 (11.46)	NR
Brochet 2008	RRMS	SDMT	Atrophy (BPF)	46	NS		NR	NR
		SDMT	Atrophy (VF)	46	NS		NR	NR
Deloire 2005	RRMS	SDMT	Atrophy (BPF)	58	NR	NR	41.19 (12.49)	45%
		SDMT	Atrophy (VF)	58	NR	NR	41.19 (12.49)	45%
Portaccio 2006	RRMS	SDMT	Atrophy (NBV)	41	NS		NR	56%
Portaccio 2009	RRMS	SDMT	Atrophy (NBV)	50	NR	NR	NR	33%

Reference	Disease Course	Cognitive Test	MRI Measure	Sample Size	*r*-value	p-value	Mean (SD) Score	Cognitively Impaired
Amato 2008	Benign MS	PASAT	Atrophy (NBV)	47	NR	NR	NR	23.4%
Hohol 1997	Chronic Progressive MS	PASAT-3	Atrophy (BICVR)	13	0.67	0.03	NR	NR
	Mixed	PASAT-3	Atrophy (BICVR)	44	0.56	<0.001	69.7 (21.3)	NR
Lazeron 2005	Mixed	PASAT-3	Atrophy (RBV)	82	0.34	<0.01	39.7 (18.93)	67%
		PASAT-2	Atrophy (RBV)	82	0.36	<0.01	29.44 (15.36)	67%
Rao 1989	Mixed	PASAT-3	Atrophy (VBR)	53	NS		NR	36%
		PASAT-2	Atrophy (VBR)	53	NS		NR	36%
Edwards 2001	Mixed	PASAT	Atrophy (normalized total cerebral hemisphere volume)	40	NR		NR	NR

**Reference**	**Disease Course**	**Cognitive Test**	**MRI Measure**	**Sample Size**	***r*-value**	**p-value**	**Mean (SD) Score**	**Cognitively Impaired**
		PASAT	Atrophy (VV)	40	NR		NR	NR
van Buchem 1998	Mixed	PASAT	Atrophy (BV)	33	0.47	0.002	NR	54%
Christodoulou 2003	Mixed	PASAT-3	Atrophy (central)	37	-0.462	=0.01	29.1 (10.1)	100%*
		PASAT-2	Atrophy (central)	37	-0.456	=0.01	39.3 (13.6)	100%*
Benedict 2007	Mixed	PASAT	Atrophy (BPF)	60	0.24	>0.05	NR	61.7%
Sumowski 2009	Mixed	PASAT + SDMT	Atrophy (TVW) low cognitive function	NR	-0.79	<0.001	NR	NR
		PASAT + SDMT	Atrophy (TVW) All patients	38	-0.46	<0.01	NR	NR
		PASAT + SDMT	Atrophy (TVW) high cognitive function	NR	-0.16	>0.5	NR	NR

Reference	Disease Course	Cognitive Test	MRI Measure	Sample Size	*r*-value	p-value	Mean (SD) Score	Cognitively Impaired
Brass 2006	Mixed	PASAT	Atrophy (BPF)	33	NS		NR	NR
Houtchens 2007	Mixed	mean of PASAT-2 and -3	Atrophy (TVW)	31	-0.590	<0.0001	35.1 (14)	NR
		mean of PASAT-2 and -3	Atrophy (BPF)	31	0.585	<0.0001	35.1 (14)	NR
Benedict 2005	Mixed	PASAT	Atrophy (LVV) – age covariate	31	0.42	0.3		NR
		PASAT	Atrophy (LVV) – age and BDI covariate	31	0.47	0.2		NR
Cortese 2011	Mixed	PASAT-3	Atrophy (SBV)	51	NS		NR	NR
Amato 2010	RRMS	mean of PASAT-2 and -3	Atrophy (PBVC)	41	NS			43.9%
Bonnet 2006	RRMS, low education	PASAT-2	Atrophy (BPF)	19	<0.30	NR	25.1 (16.26)	NR

Reference	Disease Course	Cognitive Test	MRI Measure	Sample Size	*r*-value	p-value	Mean (SD) Score	Cognitively Impaired
	group	PASAT-2	Atrophy (VF)	19	<0.30	NR	25.1 (16.26)	NR
	RRMS, high education group	PASAT-2	Atrophy (BPF)	24	<0.30	NR	37.62 (8.98)	NR
		PASAT-2	Atrophy (VF)	24	<0.30	NR	37.62 (8.98)	NR
	RRMS, low education group	PASAT-3	Atrophy (BPF)	19	<0.30	NR	36.31 (16.46)	NR
		PASAT-3	Atrophy (VF)	19	<0.30	NR	36.31 (16.46)	NR
	RRMS, high education group	PASAT-3	Atrophy (VF)	24	<0.30	NR	47.41 (8.63)	NR
		PASAT-3	Atrophy (BPF)	24	>0.40	<0.05	47.41 (8.63)	NR
Brochet 2008	RRMS	PASAT	Atrophy (BPF)	46	NS			NR
		PASAT	Atrophy (VF)	46	NS			NR
Deloire 2005	RRMS	PASAT-2	Atrophy (BPF)	58	NR	NR	32.70 (13.35)	45%
		PASAT-2	Atrophy (VF)	58	NR	NR	32.70 (13.35)	45%

Reference	Disease Course	Cognitive Test	MRI Measure	Sample Size	*r*-value	p-value	Mean (SD) Score	Cognitively Impaired
		PASAT-3	Atrophy (BPF)	58	NR	NR	42.43 (13.57)	45%
		PASAT-3	Atrophy (VF)	58	NR	NR	42.43 (13.57)	45%
Hildebrandt 2006	RRMS	PASAT-3	Atrophy (VF)	45	NR	0.8564	NR	NR
		PASAT-3	Atrophy (BPF)	45	NR	0.9423	NR	NR
Khalil 2011	RRMS	PASAT-3+SDMT	Atrophy (normalized ventricular volume)	80	-0.3	<0.01	NR	16.3%
		PASAT-3+SDMT	Atrophy (NBV)	80	0.4	<0.001	NR	13.6%
Locatelli 2004	RRMS	PASAT	Atrophy (BPF)	39	0.3	0.045	44.9 (14.9)	NR
Portaccio 2006	RRMS	PASAT-2	Atrophy (NBV)	41	NS		NR	56%
		PASAT-3	Atrophy (NBV)	41	NS		NR	56%

Reference	Disease Course	Cognitive Test	MRI Measure	Sample Size	*r*-value	p-value	Mean (SD) Score	Cognitively Impaired
Portaccio 2009	RRMS	PASAT-2	Atrophy (NBV)	50	NR		NR	33%
		PASAT-3	Atrophy (NBV)	50	NR		NR	33%
Mineev 2009	RRMS	PASAT-3	Atrophy (CPV index)	65	0.4	<0.05	NR	NR
Benedict 2006	SPMS	PASAT	Atrophy (TVW)	NR	-0.45	<0.05	NR	NR

* Mild cognitive impairment

BDI = Beck Depression Inventory; BICVR = Brain to intracranial volume ratio; BPF = Brain parenchymal fraction; BV = Brain volume; CPMS = Chronic progressive multiple sclerosis; CPV = Cerebral perfusion volume; LVV = Lateral ventricle volume; MS = Multiple sclerosis; NBV = Normalized brain volume; NR = Not reported; NS = Non-significant; PASAT = Paced auditory serial addition test; PBVC = Percentage brain volume change; PPMS = Primary progressive multiple sclerosis; RRMS = Relapse-remitting multiple sclerosis; SD = Standard deviation; SDMT = Symbol Digit Modalities Test; SPMS = Secondary progressive multiple sclerosis; TVW = Third ventricular width; VBR = Ventricle-to-brain ratio; VF = Ventricular fraction

Table 3: **Supplementary Table 3 MS Studies Evaluating Correlations between both SDMT and PASAT with MRI Measures**

Reference	Disease Course	Cognitive Test	MRI Measure	Sample Size	*r*-value	p-value	Mean ± SD Score	Cognitively Impaired
Achiron 2003	Mixed	PASAT-3	T2LV	67	NS		NR	NR
		SDMT	T2LV	67	NS		NR	NR

Reference	Disease Course	Cognitive Test	MRI Measure	Sample Size	*r*-value	p-value	Mean ± SD Score	Cognitively Impaired
Amato 2008	Benign MS	PASAT	T2LV	47	NR	NR	NR	23.4%
		SDMT	T2LV	47	NR	NR	NR	23.4%
Amato 2010	RRMS	PASAT-3	T2LV	49	NS		39.7 (12.4)	30.6%
		SDMT	T2LV	49	NS		48.7 (13.3)	30.6%
Benedict 2007	Mixed	PASAT	T2LV	60	-0.23	>0.05	NR	61.7%
		SDMT	T2LV	60	-0.45	<0.01	NR	61.7%
Bonnet 2006	RRMS	PASAT-3	T2LV	24	>0.40	<0.05	47.41 (8.63)	NR
		SDMT	T2LV	24	>0.40	<0.05	46.33 (11.46)	NR
Brass 2006	Mixed	PASAT	T2LV	33	NS		NR	NR
		SDMT	T2LV	33	NS		NR	NR
Brochet 2008	RRMS	PASAT	T2LV	46	NS		NR	NR
		SDMT	T2LV	46	-0.31	0.032	z score	NR
Christodoulou 2003	Mixed	PASAT-3	T2LV	37	-0.504	=0.01	39.3 (13.6)	100%*
		PASAT-2	T2LV	37	-0.309	=0.1	29.1 (10.1)	100%*
		SDMT	T2LV	37	-0.637	=0.01	47.0 (14.1)	100%*
Deloire 2005	RRMS	PASAT-3	T2LV	58	-0.34	<0.05	42.43 (13.57)	45%
		SDMT	T2LV	58	-0.51	<0.0001	41.19 (12.49)	45%

Reference	Disease Course	Cognitive Test	MRI Measure	Sample Size	*r*-value	p-value	Mean ± SD Score	Cognitively Impaired
Hohol 1997	CPMS	PASAT-3	T2LV	13	-0.26	0.47	NR	NR
	Mixed	PASAT-3	T2LV	44	-0.58	<0.001	69.7 (21.3)	NR
	CPMS	SDMT	T2LV	13	-0.89	<0.001	NR	NR
	Mixed	SDMT	T2LV	44	-0.66	<0.001	41.5 (11.6)	NR
Lazeron 2000	Mixed	PASAT	T2LV	39	NS		72.3 (27.2)	21%
		SDMT	T2LV	39	NS		44.9 (14.4)	21%
Lazeron 2005	Mixed	PASAT-3	T2LV	82	-0.405	<0.001	39.7 (18.93)	67%
		SDMT	T2LV	82	-0.503	<0.001	45.22 (13.67)	67%
Patti 2009	RRMS	PASAT-3	T2LV	327	-0.113	0.057		22%
		PASAT-2	T2LV	327	-0.103	0.083		22%
		SDMT	T2LV	327	-0.219	<0.001		22%
Portaccio 2006	RRMS	PASAT-2	T2LV	41	NS		NR	56%
		PASAT-3	T2LV	41	NS		NR	56%
		SDMT	T2LV	41	NS		NR	56%
Rovaris 2002	RRMS	PASAT	T2LV	34	<0.30		NR	26.5%
		SDMT	T2LV	34	-0.49	0.003	NR	26.5%
Penny 2010	PPMS	PASAT-3	T2LV	31	NS		47.13 (14.34)	29%
		SDMT	T2LV	31	-0.799	0.001	NR	29%

Reference	Disease Course	Cognitive Test	MRI Measure	Sample Size	*r*-value	p-value	Mean ± SD Score	Cognitively Impaired
Camp 2005	PPMS	Change in PASAT-2	T2LV	73	NS			26% at year 2
		Change in PASAT-3	T2LV	73	-0.303	0.012	38.28 (17.27) at year 2	26% at year 2
		Change in SDMT	T2LV	73	-0.266	0.017	40.55 (14.32) at year 2	26% at year 2
Amato 2008	Benign MS	PASAT	Atrophy	47	NR	NR	NR	23.4%
		SDMT	Atrophy	47	NR	NR	NR	23.4%
Benedict 2007	Mixed	PASAT	Atrophy	60	0.24	>0.05	NR	61.7%
		SDMT	Atrophy	60	0.61	<0.01	NR	61.7%
Brass 2006	Mixed	PASAT	Atrophy	33	NS		NR	NR
		SDMT	Atrophy	33	0.565	NR	NR	NR
		PASAT-2	Atrophy	37	-0.462	0.01	29.1 (10.1)	100%*
		PASAT-3	Atrophy	37	-0.456	0.01	39.3 (13.6)	100%*
		SDMT	Atrophy	37	-0.7	0.01	47.0 (14.1)	100%*

Reference	Disease Course	Cognitive Test	MRI Measure	Sample Size	*r*-value	p-value	Mean ± SD Score	Cognitively Impaired
Hohol 1997	Mixed: RRMS, CPMS, SPMS, PPMS	Change in PASAT-3	Atrophy	42	0.25	0.1	NR	NR
	Mixed: CPMS, SPMS, and PPMS	Change in PASAT-3	Atrophy	13	0.54	0.1	NR	NR
	Mixed: RRMS, CPMS, SPMS, PPMS	Change in SDMT	Atrophy	44	0.31	0.05	NR	NR
	Mixed: RRMS, CPMS, SPMS, PPMS	PASAT-3	Atrophy	44	0.56	<0.001	69.7 (21.3)	NR
	Mixed: CPMS, SPMS, and PPMS	PASAT-3	Atrophy	13	0.67	0.03	NR	NR
	Mixed: RRMS, CPMS, SPMS, PPMS	SDMT	Atrophy	44	0.63	<0.001	41.5 (11.6)	NR
	Mixed: CPMS, SPMS, and PPMS	SDMT	Atrophy	13	0.73	0.02	NR	NR
	RRMS	Change in PASAT-3	Atrophy	13	0.64	0.07	NR	NR
	RRMS	Change in SDMT	Atrophy	14	0.75	0.01	NR	NR
	RR-P MS	Change in PASAT-3	Atrophy	11	0.83	0.02	NR	NR

Reference	Disease Course	Cognitive Test	MRI Measure	Sample Size	*r*-value	p-value	Mean ± SD Score	Cognitively Impaired
Houtchens 2007	Mixed	mean of PASAT-2 and -3	Atrophy (TVW)	31	-0.443	0.007	35.1 (14)	NR
		mean of PASAT-2 and -3	Atrophy (BPF)	31	0.59	<0.0001	35.1 (14)	NR
		SDMT	Atrophy (TVW)	31	-0.44	0.007	48.2 (19)	NR
		SDMT	Atrophy (BPF)	31	0.57	<0.001	48.2 (19)	NR
Lazeron 2005	Mixed	SDMT	Atrophy	82	0.537	<0.001	45.22 (13.67)	67%
van Buchem 1998	Mixed	PASAT	Atrophy	33	0.47	0.002	NR	54%
		SDMT	Atrophy	33	0.24	0.14	NR	54%
Cortese 2011	Mixed	Change in PASAT-3 (1-2 years)	Atrophy	51	NS		NR	NR
		Change in SDMT (1-2 years)	Atrophy	51	NS		NR	NR
		PASAT-3	Atrophy	51	NS		NR	NR
		SDMT	Atrophy	51	0.39	0.006	NR	NR
Amato 2010	RRMS	mean of PASAT-2 and -3	Atrophy	41	NS		NR	43.9%
		SDMT	Atrophy	41	NS		NR	43.9%

Reference	Disease Course	Cognitive Test	MRI Measure	Sample Size	*r*-value	p-value	Mean ± SD Score	Cognitively Impaired
Bonnet 2006	RRMS, low education group	PASAT-2	Atrophy (BPF)	19	<0.30	NR	25.1 (16.26)	NR
		PASAT-2	Atrophy (VF)	19	<0.30	NR	25.1 (16.26)	NR
	RRMS, high education group	PASAT-2	Atrophy (BPF)	24	<0.30	NR	37.62 (8.98)	NR
		PASAT-2	Atrophy (VF)	24	<0.30	NR	37.62 (8.98)	NR
	RRMS, low education group	PASAT-3	Atrophy (BPF)	19	<0.30	NR	36.31 (16.46)	NR
		PASAT-3	Atrophy (VF)	19	<0.30	NR	36.31 (16.46)	NR
	RRMS, high education group	PASAT-3	Atrophy (VF)	24	<0.30	NR	47.41 (8.63)	NR
		PASAT-3	Atrophy (BPF)	24	>0.40	<0.05	47.41 (8.63)	NR
	RRMS, low education group	SDMT	Atrophy (VF)	19	<0.30	NR	36.31 (8.49)	NR
		SDMT	Atrophy (BPF)	19	<0.30	NR	36.31 (8.49)	NR
	RRMS, high education group	SDMT	Atrophy (VF)	24	<0.30	NR	46.33 (11.46)	NR
		SDMT	Atrophy (BPF)	24	>0.40	<0.05	46.33 (11.46)	NR
Brochet 2008	RRMS	PASAT	Atrophy (BPF)	46	NS		NR	NR
		PASAT	Atrophy (VF)	46	NS		NR	NR
		PASAT	Atrophy(BPF)	46	NS		NR	NR
		PASAT	Atrophy (VF)	46	NS		NR	NR
		PASAT	Atrophy (BPF)	46	NS		NR	NR
		PASAT	Atrophy (VF)	46	NS		NR	NR
		SDMT	Atrophy (BPF)	46	NS		NR	NR

Reference	Disease Course	Cognitive Test	MRI Measure	Sample Size	*r*-value	p-value	Mean ± SD Score	Cognitively Impaired
		SDMT	Atrophy (VF)	46	NS		NR	NR
		SDMT	Atrophy (VF)	46	NS	NR	NR	NR
		SDMT	Atrophy (BPF)	46	NS		NR	NR
		SDMT	Atrophy (VF)	46	NS		NR	NR
		SDMT	Atrophy (BPF)	46	0.5	<0.001	z score	NR
Deloire 2005	RRMS	PASAT-2	Atrophy(BPF)	58	NR	NR	32.70 (13.35)	45%
		PASAT-2	Atrophy (VF)	58	NR	NR	32.70 (13.35)	45%
		PASAT-3	Atrophy (BPF)	58	NR	NR	42.43 (13.57)	45%
		PASAT-3	Atrophy (VF)	58	NR	NR	42.43 (13.57)	45%
		SDMT	Atrophy (BPF)	58	NR	NR	41.19 (12.49)	45%
		SDMT	Atrophy (VF)	58	NR	NR	41.19 (12.49)	45%
Portaccio 2006	RRMS	PASAT-2	Atrophy	41	NS		NR	56%
		PASAT-3	Atrophy	41	NS		NR	56%
		SDMT	Atrophy	41	NS		NR	56%
Portaccio 2009	RRMS	PASAT-2	Atrophy	50	NR	NR	NR	33%
		PASAT-3	Atrophy	50	NR	NR	NR	33%
		SDMT	Atrophy	50	NR	NR	NR	33%

*Mild cognitive impairment

BPF = Brain parenchymal fraction; CPMS = Chronic progressive multiple sclerosis; MS = Multiple sclerosis; NBV = Normalized brain volume; NR = Not reported; NS = Non-significant; PASAT = Paced auditory serial addition test; PPMS = Primary progressive multiple sclerosis; RRMS = Relapse-remitting multiple sclerosis; SD = Standard deviation;

SDMT = Symbol Digit Modalities Test; SPMS = Secondary progressive multiple sclerosis ; T2LV = T2-weighted lesion volume; TVW = Third ventricular width; VF = Ventricular fraction
